# The Effect of Chunghyul-Dan on Hyperventilation-Induced Carbon Dioxide Reactivity of the Middle Cerebral Artery in Normal Subjects: A Dose-Dependent Study

**DOI:** 10.1155/2017/4567217

**Published:** 2017-04-20

**Authors:** Chul Jin, Sang-Kwan Moon, Seung-Yeon Cho, Seong-Uk Park, Woo-Sang Jung, Jung-Mi Park, Chang-Nam Ko, Ki-Ho Cho, Seungwon Kwon

**Affiliations:** Department of Cardiology and Neurology, College of Korean Medicine, Kyung Hee University, Seoul, Republic of Korea

## Abstract

*Background*. This study was conducted to show the prompt effect of chunghyul-dan (CHD) on cerebral hemodynamics in order to provide evidence for its use in stroke prevention.* Methods*. Hyperventilation-induced CO_2_ reactivity of the middle cerebral artery was measured in 12 healthy male volunteers (mean age: 26.3 ± 1.1 years) using transcranial Doppler sonography. All subjects were examined before and for 3 hours after administration, with an interval of 1 week between measurements.* Results*. Compared to baseline, the CO_2_ reactivity of the middle cerebral artery increased significantly at 2 and 3 hours after the administration of CHD (600 mg and 1200 mg). The mean blood pressure and heart rate did not vary from the baseline values in all groups.* Conclusion*. These data suggest that CHD administration (especially 600 mg) immediately improves cerebral blood flow.

## 1. Introduction

Chunghyul-dan (CHD) is an herbal complex containing 80% ethanol extract and is composed of Scutellariae Radix, Coptidis Rhizoma, Phellodendri Cortex, Gardeniae Fructus, and Rhei Rhizoma. Previous studies have reported that CHD has antihyperlipidemic effects [1, 2]; improves arterial stiffness [3]; and has anti-inflammatory [4, 5], antioxidant [6], antihypertensive [7], and neuronal protective [8–10] effects. Furthermore, CHD reportedly results in nitric oxide synthase (NOS) mRNA expression in endothelial cells [11], which may affect NO endothelial function. In addition, owing to its inhibitory effects on the recurrence of small vessel occlusion-type cerebral infarction [12], CHD has been used as an ischemic stroke preventive agent.

Small vessel occlusion-type cerebral infarction is caused by blood-brain barrier damage induced by cerebral endothelial dysfunction [13], impaired cerebral autoregulation (CA) [14], and prothrombotic changes [15]. Among them, CA is largely attributed to two factors: blood pressure variability [16] and carbon dioxide (CO_2_) cerebrovascular reactivity (CVR) [17]. However, cerebral blood flow remains constant at a mean blood pressure of 60–150 mmHg, while it is immediately sensitive to the concentration of CO_2_. Therefore, CVR is an important factor in the control of CA. CVR might be reduced in various pathological conditions such as hypertension [18], diabetes mellitus [19, 20], heart failure [21], arterial stenosis or occlusion [22], aging [23], cognitive impairment [24], depression [25], Alzheimer's disease [26], and white matter degeneration of the brain [27]. Furthermore, decreased CVR is known to increase the risk of ischemic stroke [28, 29]. In a previous meta-analysis, the usefulness of CVR for prediction of ischemic stroke in carotid artery stenosis and occlusion was evaluated. Seven hundred fifty-four patients (9 prospective studies) were included and impaired CVR was independently associated with an increased risk of ischemic stroke (*p* < 0.0001) [28].

The inhibitory effect of CHD on the recurrence of small vessel occlusion-type cerebral infarction is believed to be due to the control of stroke risk factors and improved endothelial function. However, previous studies suggested that systemic and cerebral endothelial functions are independent of each other [30, 31]. Thus, it is unclear whether CHD could also affect cerebral endothelial function. In addition, it remains undetermined whether CHD directly affects cerebral blood flow. Therefore, it is difficult to explain the inhibitory effect of CHD on the recurrence of ischemic stroke. Thus, it is necessary to measure the CVR, which is a predictor of the risk of ischemic stroke and an important factor in the control of cerebral blood flow, in order to explain the inhibitory effect of CHD on the recurrence of small vessel occlusion-type cerebral infarction. CVR was assessed as the degree of cerebral blood flow change with CO_2_ or acetazolamide injection, which is a vasoactive factor. CVR is directly and indirectly measured using transcranial Doppler sonography (TCD), blood oxygen level-dependent functional magnetic resonance imaging (BOLD fMRI), single photon emission computed tomography (SPECT), and positron emission tomography (PET). Among them, TCD is widely used because it is relatively easy and economical to measure CVR using this noninvasive method.

Thus, we assumed that administration of CHD would directly increase the CVR and prevent small vessel occlusion-type cerebral infarction. To confirm this hypothesis, we observed cerebral hemodynamics before and after the administration of CHD via hyperventilation-induced CO_2_ reactivity and corrected cerebrovascular velocity (correction of end-tidal CO_2_ partial pressure to 40 mmHg) measured using TCD. Although hypercapnia CVR has larger evidences on stroke risk, we used hyperventilation-induced CO_2_ reactivity to minimize the influence of concentration of CO_2_ on cerebral blood flow during study protocol. In addition, we measured CVR for 300 mg, 600 mg, and 1200 mg doses of CHD and no-use in order to determine the optimal dose of CHD.

## 2. Materials and Methods

### 2.1. Subjects

Twelve healthy male volunteers (mean age: 26.3 ± 1.1 (SD; standard deviation) years) were recruited. The subjects had no history of cerebrovascular disease, heart diseases, hypertension, diabetes mellitus, thyroid disease, or psychiatric problems. All subjects were prohibited from consuming alcohol, coffee, smoking, and any medications for 12 hours before the trial.

### 2.2. CHD Preparation

The CHD was manufactured by the Pharmaceutical Department of Kyung Hee University Korean Medicine Hospital. Total of 1.2 g of Scutellariae Radix, Coptidis Rhizoma, Phellodendri Cortex, Gardeniae Fructus, and Rhei Rhizoma was extracted by refluxing with 15-fold volume of 80% ethanol for 2 hours, filtered, and concentrated under reduced pressure to reduce the water content to 50%. The detailed component of CHD is shown in Table 1.

### 2.3. Study Design

This study employed an open-label, randomized, and multiple-crossover trial design. The subjects visited four times at 8 AM after fasting. At the first visit (visit 1), only water was administered to the subjects. In subsequent visits (visits 2–4), three doses of CHD (300, 600, or 1200 mg) were randomly administered to the subjects. In addition, the intervals between each visit were least 1 week in order to minimize the residual effects of the CHD.

### 2.4. Assessment Methods

The cerebral blood flow velocity and CVR of the middle cerebral artery were measured using a Multi-Dop X4 cerebral blood flow ultrasound (transcranial Doppler) system (Compumedics DWL, Singen, Germany) as described previously [32–34]. At each visit, the cerebral blood flow velocity of the middle cerebral artery was measured in the unilateral temporal window using a 2 MHz ultrasonic probe with the participant in a supine position. The probe was fixed to the temporal window using a detachable bilateral probe holder (LAM-Rack; Compumedics DWL). The depth for detecting the middle cerebral artery was 45 to 60 mm and the best waveforms were recorded. After stabilization for five minutes before measurement, the mean cerebral blood flow velocity was recorded, followed by a four-minute rest and one-minute hyperventilation. The mean cerebral blood flow velocities in the middle cerebral artery were measured at rest in the normocapnic state and in the hypocapnic state during hyperventilation. All TCD waveforms were stored in the computer for analysis.

CVR is the percentage difference in mean cerebral blood flow velocity according to the change of *P*_ETCO_2__ and is expressed as %/min according to the following formula:

(1) $$\begin{array}{c} CO_{2}\text{ reactivity}=100\times \frac{\left[V_{rest}-V_{hypocapnia}\right]/V_{rest}}{{\Delta P}_{{ETCO}_{2}}}, \end{array}$$

where *V*_rest_ is blood flow velocity at rest (the most stable 10 seconds measured), *V*_hypocapnia_ is blood flow velocity during hyperventilation (the most stable 10 seconds measured in the second half of 30 seconds), and Δ*P*_ETCO_2__ is difference in *P*_ETCO_2__ between the two periods.

A corrected cerebral blood flow velocity (CV40, cm/s) was used, which was converted into a 40 mmHg CO_2_ concentration according to a previously suggested formula [35] because cerebral blood flow velocity is affected by the blood concentration of CO_2_.

(2) CV40  Corrected  Velocity  at  PETCO2  40 mmHg=V1·ebPCO2  40 mmHg-P1CO2

*b* is CO_2_ reactivity,* V*_1_ is blood flow velocity of* P*1CO2, and *P*_ETCO2_ is end-tidal CO_2_ partial pressure.

To measure and control for variables such as blood pressure, pulse rate, and expiratory CO_2_ concentration, various modules of the Cardiocap S/5 collector (Datex-Ohmeda, Helsinki, Finland) were used in a quiet room maintained at a constant temperature. Blood pressure and pulse rate were measured before hyperventilation. Blood pressure was recorded as mean blood pressure measured 3 times at 2-minute intervals with a cuff wrapped around the left upper arm; pulse rate was measured continuously using an oximeter placed on the right finger of the subject. Nasal prongs connected to the Cardiocap S/5 collector were placed in the nose of the subject. During the trial, the subjects were instructed to breathe only with nasal breathing and CO_2_ concentration was monitored during expiration. The pulse rate and CO_2_ concentration values stored in the Cardiocap S/5 collector program were averaged over a specific time using the snapshot function. The above three parameters were continuously monitored during the trial and stored on a computer connected to the Cardiocap S/5 collector.

**Figure 1:**
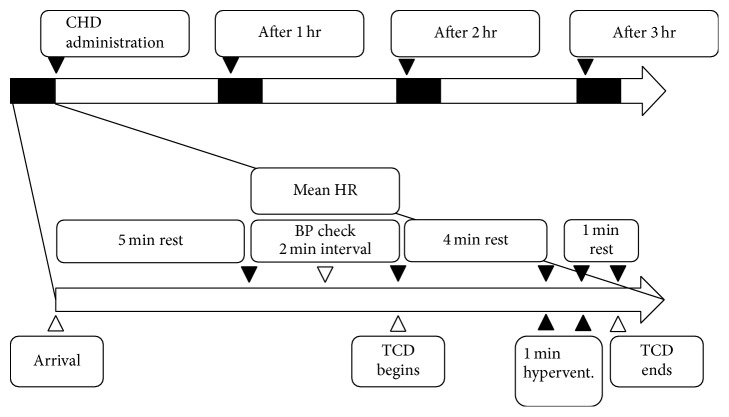
Study design timeline. Measurements were repeated four times, at least 1 week apart, according to the multiple-crossover design. CHD, chunghyul-dan; hr, hour; HR, heart rate; BP, blood pressure; TCD, transcranial Doppler sonography; hypervent., hyperventilation.

We used these measuring devices to repeatedly measure CO_2_ reactivity, CV40, and blood pressure at 1, 2, and 3 hours after the oral administration. Each measurement point required approximately 15 minutes to complete; during this time, the above devices were attached in order to record and store the measured parameters (Figure 1).

### 2.5. Statistical Analysis

The statistical analyses in this study were performed using Statistical Package for the Social Sciences version 12.0 for Windows (SPSS, Inc., Chicago, IL). All data were expressed as mean ± standard deviation (SD). The changes according to the time and dose after the administration of CHD or water were analyzed by two-way repeated measures analysis of variance (ANOVA). In addition, one-way repeated measures ANOVA with Bonferroni correction was used for comparison (at the same time or the same dose). Finally, correlations between CVR and CV40 were assessed by Pearson's correlation analysis. *p* values less than 0.05 were considered statistically significant.

## 3. Results

### 3.1. CVR (%/min)

Two-way ANOVA with repeated measures showed a significant interaction with time × treatment (*F* = 10.720, *p* < 0.001). In Bonferroni post hoc analysis, the 600 mg group showed significant differences compared with the control and 300 mg groups (*p* = 0.024 and 0.023, resp.). Furthermore, the 1200 mg group also showed significant differences compared with the control and 300 mg groups (*p* = 0.034 and 0.044, resp.).

There was no significant difference in the one-way repeated measures ANOVA in the control and 300 mg CHD groups. However, there were significant interactions with time (*p* < 0.001) in the groups given 600 and 1200 mg of CHD. Pos thoc analysis revealed statistically significant differences at 1 (T1, *p* = 0.001), 2 (T2, *p* < 0.001), and 3 (T3, *p* < 0.001) hours after administration of 600 mg CHD compared to the before CHD administration (T0). There were also statistically significant differences at 2 (*p* = 0.002) and 3 (*p* < 0.001) hours after administration of 1200 mg CHD compared to the before CHD administration (T0) (Table 2).

**Figure 2:**
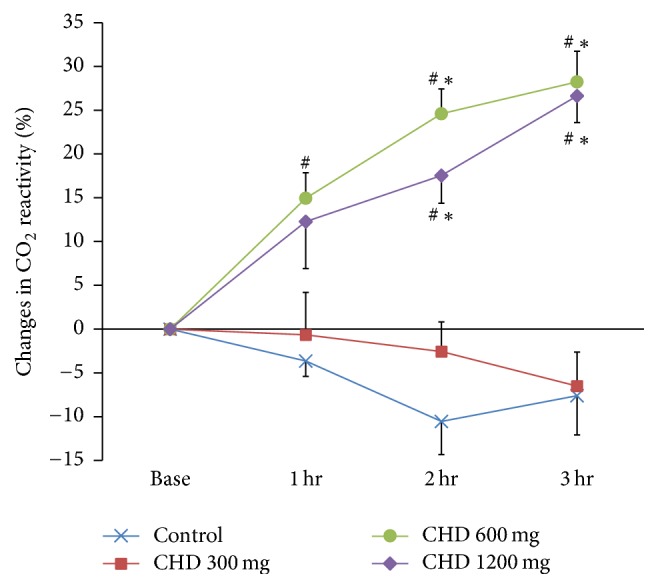
Percent changes in CO_2_ reactivity (%) before and after CHD administration. The lines represent the means ± SEM. CHD, chunghyul-dan; hr, hour. ^#^*p* < 0.05 versus control at the same time points by Bonferroni multiple comparisons after one-way repeated measures analysis of variation (RM ANOVA). ^*∗*^*p* < 0.05 versus CHD 300 mg at the same time points by Bonferroni multiple comparisons after one-way RM ANOVA.

The changes in CVR expressed as percent (%) of baseline value were analyzed using one-way repeated measures ANOVA with Bonferroni correction for each dose group at the same time point. In the group given 600 mg CHD, there were statistically significant differences compared with the control group at 1, 2, and 3 hours after administration (*p* = 0.006, *p* < 0.001, and *p* = 0.001, resp.) and there were also statistically significant differences compared with the 300 mg group at 2 and 3 hours (*p* < 0.001, and *p* < 0.001, resp.). Furthermore, there were statistically significant differences compared with the control group at 2 and 3 hours after administration in the group given 1200 mg CHD (*p* = 0.005 and *p* < 0.001). There were also statistically significant differences at 2 and 3 hours after administration compared with the group given 300 mg (*p* = 0.005, *p* < 0.001) (Figure 2).

### 3.2. Results of Corrected Cerebral Blood Flow Velocity (CV40, cm/s)

The corrected cerebral blood flow velocity showed a significant interaction (*F* = 3.227, *p* = 0.002) for time × treatment, but the difference between the treatment groups was not significant (Bonferroni post hoc test). When analyzed by treatment group, there was a significant interaction with time in the 600 mg CHD group (*p* = 0.024). However, there was no significant difference between before CHD administration (T0) and 3 hours after administration (*p* = 0.075) (Table 3).

**Figure 3:**
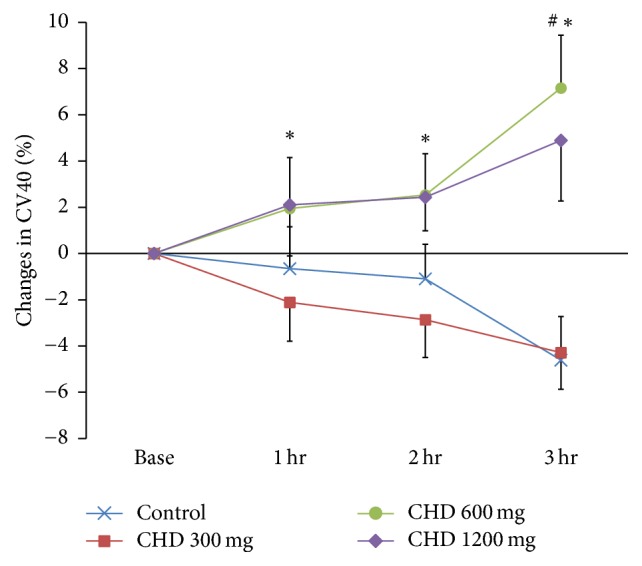
Percent changes in CV40 (%) before and after CHD administration. Values are means ± SEM. CHD, chunghyul-dan; hr, hour. ^#^*p* < 0.05 versus control at the same time points by Bonferroni multiple comparisons after one-way repeated measures analysis of variation (RM ANOVA). ^*∗*^*p* < 0.05 versus CHD 300 mg at the same time points by Bonferroni multiple comparisons after one-way RM ANOVA.

The changes in CV40 expressed as percent (%) of baseline value were analyzed by one-way repeated measures ANOVA with Bonferroni correction for each dose group at the same time point. The CV40 value at 3 hours after the administration (T3) of 600 mg CHD was significantly different from that of the control group (*p* = 0.030). The CV40 values of the 600 mg CHD group were significantly different from those of the 300 mg group at 1, 2, and 3 hours (*p* = 0.023, 0.034, and 0.001, resp.) (Figure 3).

### 3.3. Results of Mean Blood Pressure and Mean Heart Rate

There was no significant difference in mean blood pressure and mean heart rate before and after the administration of CHD (all doses) (Table 4).

### 3.4. Correlation between Changes in CVR and Corrected Cerebral Blood Flow Velocity with Administration of CHD

**Figure 4:**
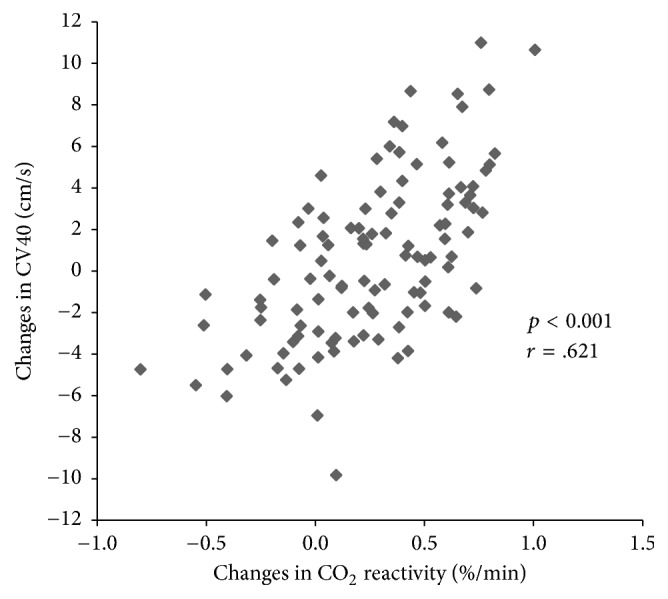
Pearson's correlation analysis of the changes in CO_2_ reactivity and CV40 before and after CHD administration. A significant correlation is shown between CHD administration-mediated changes in CO_2_ reactivity and the CV40 of the MCA (*p* < 0.001, *r* = 0.621).

A significant correlation was observed between the changes of CVR and corrected cerebral blood flow velocities in subjects who were given CHD (Figure 4).

## 4. Discussion

The CVR and corrected cerebral blood flow velocity (CV40) of the middle cerebral artery measured before and after the administration of CHD (600 and 1200 mg) showed a continuous increase over time. Mean blood pressure and heart rate did not significantly differ before and after the administration of CHD. In addition, no participant complained of adverse effects after taking CHD.

Hyperventilation-induced CO_2_ CVR significantly increased after administration of 600 and 1200 mg CHD. CHD inhibits 3-hydroxy-3-methylglutaryl-coenzyme A (HMG-CoA) reductase [1]; thus, it may decrease the total cholesterol and low-density lipoprotein- (LDL) cholesterol levels [2]. Furthermore, CHD upregulates mRNA expression of endothelial NOS (eNOS) and leads to the production of NO in vascular endothelial cells [11]. In previous studies, CHD showed anti-inflammatory effects by reducing NO induced by cyclooxygenase-2 (COX-2), interferon gamma (IFN-r), interleukin 4 (IL-4), tumor necrosis factor alpha (TNF-a), prostaglandin E2 (PGE-2), vascular cell adhesion molecule 1 (VCAM-1), monocyte chemoattractant protein-1 (MCP-1), and inducible NOS (iNOS) [4–6]. CHD also showed vascular endothelial protective effects such as antiapoptosis, cell cycle progression, induced cell migration, and proliferation in vascular endothelial cells [36]. Among these various effects of CHD, the action of producing NO in eNOS could improve cerebrovascular endothelial function and increase CVR. Similar studies reported the effects of statin on endothelial NO synthesis and enhancement of CVR. Kaesemeyer et al. [37] reported that statins rapidly activate eNOS in bovine aortic endothelial cells and lead to the production of NO. Sterzer et al. [38] suggested that improvement of CVR after statin administration in small vessel occlusion-type ischemic stroke patients appears with the upregulation of eNOS and blood flow control, which is more reactive to NO. Furthermore, Sander et al. reported that CVR could be also increased by short-term statin administration in healthy subjects [39]. Based on these results, we assumed that the mechanism of CHD on CVR in healthy subjects is similar to the effects of statins. Since CVR decreases after acute stroke and decreased CVR increases the risk of stroke and TIA, the use of CHD could increase CVR and prevent stroke.

Corrected cerebral blood flow velocity (CV40) increased significantly after the administration of 600 mg CHD compared to no treatment. This study measured mean blood pressure, heart rate, and *P*_ETCO2_, which could affect the cerebral blood flow, but no statistically significant differences were found compared with the resting (normocapnic) condition.

We measured CVR and cerebral blood flow velocity with transcranial Doppler to indirectly estimate the changes in cerebral blood flow. The following research results provide theoretical background to these measurement methods. Markwalder et al. [35] reported that the change in cerebral blood flow velocity could be a change in cerebral blood flow, since the cerebral blood flow-*P*CO_2_ response curve was similar to that of the cerebral blood flow velocity-*P*_ETCO2_, assuming no change in vessel diameter. Sorteberg et al. [40] suggested that cerebral blood flow velocity and local cerebral blood flow were statistically correlated. Lindegaard et al. [41] reported a significant correlation between the cerebral artery flow velocity and internal carotid artery blood flow. Therefore, we suggest that the administration of 600 mg CHD may increase cerebral blood flow. Based on these results, CHD may improve lowered cerebral blood flow states such as hypertension, ischemic stroke, Alzheimer's disease [42, 43], cerebral atrophy, and aging [44, 45].

The present study protocol was not designed to reveal the mechanism. Therefore, the mechanism of the immediate effect of CHD on cerebrovascular reactivity and cerebral blood flow velocity could not be clearly proven. However, the presumed mechanism is as follows. Control of cerebral blood flow through changes in vessel diameter can be divided into endothelium-dependent and endothelium-independent mechanisms. Endothelium-independent vasodilation occurs when NO produced by nonendothelial cells acts directly on vascular smooth muscle or when vascular smooth muscle relaxation occurs because of Ca^2+^ channel inhibition. In normal subjects, changes in cerebral blood flow were not observed after the administration of sodium nitroprusside (SNP) [46, 47] or nitroglycerin [48], which produces NO. In addition, cerebral blood flow velocity was increased when nimodipine, a calcium channel blocker, was administered to patients with subarachnoid hemorrhage [49]. However, there was no significant change in cerebral blood flow velocity and CVR was decreased when nimodipine was administered to healthy subjects [50]. These results suggest that endothelium-independent blood flow regulation has little effect on cerebral blood flow velocity and CVR. Therefore, it is unlikely that the effect of CHD on cerebral blood flow is mediated by an endothelium-independent regulation mechanism, and further studies are needed.

Endothelium-dependent cerebral blood flow regulation is the result of variation of the diameter of vessels due to NO, a vasodilator secreted by vascular endothelial cells, or Endothelin-1, a vasoconstrictor. NO has several effects, including vasodilation, increased blood flow, decreased vessel resistance, decreased blood pressure, inhibition of platelet aggregation and adhesion, antioxidant, and vascular endothelial cell protection [51–53]. Basal NO, which is continuously secreted by the endothelial cells of the cerebral vessels, plays an important role in regulating cerebral blood flow and vascular tone [54]. Cerebral blood flow velocity reportedly increases when L-arginine, which increases NO production in endothelial cells, is administered to normal subjects [55]. In another study, cerebral blood flow velocity did not change after administration of L-arginine, and CVR increased only in subjects with originally decreased CVR [56]. However, administration of NG-Methyl-L-arginine acetate (L-NMMA) inhibited NO synthesis in the elderly, resulting in decreased cerebral blood flow velocity; there was no change in cerebral blood flow velocity and CVR after administration to young healthy persons [57, 58]. These results suggest that the NO increase in endothelial cells observed after the administration of CHD might affect cerebral blood flow. However, increased NO in the endothelial cells increased CVR and cerebral blood flow velocity after the administration of CHD to young healthy subjects. Furthermore, CV40 in 600 mg and 1200 mg CHD group increased over different sessions (T0 to T3) and a significant correlation was observed between the changes of CVR and CV40 in subjects who were given CHD (Figure 4). Therefore, the increased cerebral blood flow velocity could be also one of the main mechanisms of the CVR increase after CHD administration. Therefore, further studies concerned with indicators such as blood viscosity or erythrocyte deformability, which may affect cerebral microcirculation, are needed to determine the concrete mechanism of CHD on CVR.

There was no significant change in CVR and corrected cerebral blood flow velocity (CV40) after the administration of 300 mg or no CHD. However, CVR and CV40 tended to decrease with time. These results are consistent with those of Strohm et al. [59] and Conroy et al. [60], who reported that CVR in normal subjects declined from morning to evening and cerebral blood flow velocity declined from midnight to noon. In this study, CVR and cerebral blood flow velocity decreased with daily fluctuation because the study was conducted from 8:00 to 11:00 AM. This finding suggests that increased CVR and cerebral blood flow velocity after administration of 600 or 1200 mg CHD might be a more significant outcome.

The mean blood pressure and heart rate did not differ significantly following the administration of CHD (or not). Furthermore, the dose of CHD also did not affect the mean blood pressure and heart rate. A previous study showed a significant decrease in systolic blood pressure and no change in pulse rate in patients with acute stroke with hypertension after administering 1200 mg CHD daily for 2 weeks [7]. This difference in blood pressure lowering effect may be due to the single administration of CHD to normal subjects in this study; however, it also did not affect the heart rate.

The limitations of the present study are as follows: the study group contained a small number of subjects, the subjects and researchers were not blinded, and a nonplacebo group was used as a control group. However, in the control group of a previous study [33] that used the same protocol, CVR, CV40, mean blood pressure, and pulse rate of the middle cerebral artery measured after taking the placebo drug were similar to those of the untreated group in this study. Therefore, the nocebo effect may be excluded. Although it was assumed that immediate cerebral blood flow improvement after CHD administration would be an effect of NO production through the activation of eNOS in the vascular endothelial cells, there are some limitations in explaining the concrete relationship, as this study did not measure plasma NO levels. Therefore, future studies with better design are necessary to assess these issues.

## Figures and Tables

**Table 1:** Constituents of chunghyul-dan (CHD).

Components	Part used	Weight (g/capsule)
*Scutellaria baicalensis* Georgi (Labiatae)	Root	0.28
*Coptis japonica* Makino (Ranunculaceae)	Root	0.28
*Phellodendron amurense* Ruprecht (Rutaceae)	Cortex	0.28
*Gardenia jasminoides* Ellis (Rubiaceae)	Fruit	0.28
*Rheum palmatum* L. (Polygonaceae)	Root	0.07

Total		1.2

**Table 2:** Changes in hyperventilation-induced CO_2_ reactivity (%/min) before and after CHD administration.

(*n* = 12)		(T0) Before administration	After administration			*p* ^†^	*p* ^‡^	*p* ^*∗*^
			T1 (1 hr)	T2 (2 hr)	T3 (3 hr)			
Control		2.16 ± 0.42	2.08 ± 0.39	1.93 ± 0.33	1.99 ± 0.36	<0.001	—	0.090
CHD	300 mg	2.07 ± 0.34	2.06 ± 0.51	2.02 ± 0.36	1.94 ± 0.43		1.000	0.501
CHD	600 mg	2.06 ± 0.31	2.37^#^ ± 0.39	2.56^#^ ± 0.42	2.64^#^ ± 0.44		0.024	<0.001
CHD	1200 mg	2.00 ± 0.25	2.24 ± 0.28	2.35^#^ ± 0.38	2.53^#^ ± 0.30		0.034	<0.001

All data are means ± SD. CHD, chunghyul-dan; hr, hour.

^†^ *p*: time × treatment interaction effect by two-way repeated measures analysis of variation (RM ANOVA).

^‡^ *p*: post hoc test by Bonferroni multiple comparisons versus control after two-way RM ANOVA.

^*∗*^ *p*: within-measure (time) effect by one-way RM ANOVA.

^#^ *p* < 0.05 versus before administration (baseline) by Bonferroni multiple comparisons after one-way RM ANOVA.

**Table 3:** Changes in corrected blood flow velocity at *P*_ETCO2_ = 40 mmHg (CV40, cm/sec) before and after CHD administration.

(*n* = 12)		(T0) Before administration	After administration			*p* ^†^	*p* ^*∗*^
			T1 (1 hr)	T2 (2 hr)	T3 (3 hr)		
Control		54.7 ± 8.77	54.3 ± 8.87	54.1 ± 9.30	52.2 ± 9.69	0.002	0.071
CHD	300 mg	54.4 ± 7.28	53.2 ± 8.37	52.8 ± 7.62	52.1 ± 9.13		0.061
CHD	600 mg	51.4 ± 9.49	52.4 ± 10.69	52.7 ± 11.39	55.2 ± 12.92		0.024
CHD	1200 mg	52.2 ± 7.34	53.3 ± 7.53	53.5 ± 8.88	54.8 ± 7.70		0.255

Mean ± SD values are shown. CHD, chunghyul-dan; hr, hour.

^†^ *p*: time × treatment interaction by two-way repeated measures analysis of variation (RM ANOVA).

No significant difference between each group (Bonferroni multiple comparisons).

^*∗*^ *p*: time effect of individual treatment by one-way RM ANOVA.

No significant difference between individual time points (Bonferroni multiple comparisons).

**Table 4:** Changes in mean blood pressure and heart rate before and after CHD administration.

(*n* = 12)		(T0) Before administration	After administration			*p* ^†^	*p* ^*∗*^
			T1 (1 hr)	T2 (2 hr)	T3 (3 hr)		
Mean blood pressure (mmHg)							
Control		84.5 ± 4.57	84.8 ± 5.27	85.4 ± 4.81	85.2 ± 4.32	0.742	0.736
CHD	300 mg	84.3 ± 2.87	83.6 ± 3.21	83.3 ± 4.47	83.4 ± 4.67		0.695
CHD	600 mg	82.3 ± 4.16	83.6 ± 4.02	82.9 ± 5.26	83.4 ± 5.07		0.611
CHD	1200 mg	83.4 ± 4.79	84.4 ± 5.25	84.9 ± 4.02	84.7 ± 4.09		0.518

Heart rate (beats per minute)							
Control		66.7 ± 9.47	65.6 ± 8.63	64.8 ± 8.36	65.0 ± 9.47	0.791	0.154
CHD	300 mg	66.5 ± 6.24	65.8 ± 6.41	64.6 ± 7.06	65.2 ± 6.39		0.085
CHD	600 mg	65.2 ± 9.00	65.1 ± 9.79	63.7 ± 8.58	64.3 ± 7.68		0.374
CHD	1200 mg	63.7 ± 9.26	64.3 ± 7.71	63.9 ± 9.73	62.8 ± 8.64		0.356

All data are means ± SD. CHD, chunghyul-dan; hr, hour.

^†^ *p*: time × treatment interaction by two- way repeated measures analysis of variation (RM ANOVA).

^*∗*^ *p*: time effect of individual groups by one-way RM ANOVA.
